# The Relation of Moderate Alcohol Consumption to Hyperuricemia in a Rural General Population

**DOI:** 10.3390/ijerph13070732

**Published:** 2016-07-20

**Authors:** Zhao Li, Xiaofan Guo, Yamin Liu, Ye Chang, Yingxian Sun, Guangshuo Zhu, Maria Roselle Abraham

**Affiliations:** 1 Department of Cardiology, the First Hospital of China Medical University, Shenyang 110001, China; meilichian@aliyun.com (Z.L.); guoxiaofanl1986@outlook.com (X.G.); guanyufan1986@gmail.com (Y.C.); 2 Department of Pharmacy, Zhongda Hospital, Southeast University, Nanjing 210009, China; zhaolisy@outlook.com; 3 Department of Cardiology, Johns Hopkins University, Baltimore, MD 21205, USA; zhuguangshuo1976@gmail.com (G.Z.); meilichian3@gmail.com (M.R.A.)

**Keywords:** alcohol, consumption, risk factor, prevalence, hyperuricemia

## Abstract

Background: although alcohol abuse is known to increase serum uric acid, the relation between moderate drinking and uric acid have remained poorly understood. We performed this study to evaluate whether different alcohol consumption level has different effects on the risk of hyperuricemia based on a rural general population. Method: multi-stage cluster sampling method was used to select a representative sample of individuals aged 35 years or older. Participants were asked to provide information about their alcohol consumption. Data regarding the demographic and lifestyle characteristics and the blood biochemical indexes of these participants were collected by well-trained personnel. Results: in total, 11,039 participants aged 35 years or older were included (4997 men and 6042 women). The prevalence of hyperuricemia in the different male alcohol consumption groups was 11.9% in non-drinkers, 12.6% in moderate drinkers, and 16.3% in heavy drinkers (*p* < 0.001). In females, the rates were 6.3% in non-drinkers, 8.1% in moderate drinkers, and 6.6% for heavy drinkers (*p* = 0.818). In males, multivariate logistic regression analyses shows heavy drinkers had an approximately 1.7-fold higher risk of hyperuricemia (OR: 1.657, 95% CI: 1.368 to 2.007, *p* < 0.001) than non-drinkers; moderate drinkers did not experience a significant increase in risk (OR: 1.232, 95% CI: 0.951 to 1.596, *p* = 0.114)). Multivariate logistic regression analyses of females showed that, compared with non-drinkers, neither moderate nor heavy drinkers had a significantly increased risk of hyperuricemia (OR: 1.565, 95% CI: 0.521 to 4.695, *p* = 0.425 for heavy drinkers; OR: 0.897, 95% CI: 0.117 to 6.855, *p* = 0.916 for moderate drinkers). Conclusions: heavy alcohol consumption increased the risk of hyperuricemia for males but not for females. Among both males and females, moderate alcohol consumption did not increase the risk of hyperuricemia.

## 1. Introduction

Data has shown the incidence of hyperuricemia is increasing worldwide [1,2,3]. Such an epidemic has deleterious consequences on quality of life and may increase cardiovascular mortality [4,5,6,7,8]. Recent studies suggest that moderate alcohol consumption is associated with a low risk of cancer and coronary heart disease [9,10,11]. However, there is a lack of studies which evaluate the effect of moderate alcohol consumption on serum uric acid. Among 2062 healthy volunteers, Alatalo et al. reported the serum uric acid concentrations in male moderate drinkers were significantly higher, and in females they were lower, than in the corresponding groups of abstainers [12]. The report which is based on the Third National Health and Nutrition Examination Survey in America suggested that the effect of individual alcoholic beverages on serum uric acid levels varied substantially: beer conferred a larger increase than liquor, whereas moderate wine drinking did not increase serum uric acid levels [13].

Until now, few studies have focused on the relation between moderate alcohol consumption and serum uric acid level in a general population. If there is a different effect of high and moderate alcohol consumption on uric acid in a general crowd, it would have practical implications for prevention and management of hyperuricemia in the real world. Thus, we performed the present study to evaluate whether different alcohol consumption has different effects on the risk of hyperuricemia in a general population from rural China, with the aim of providing evidence that may be useful for the prospective study on management of individuals with different alcohol consumption rates.

## 2. Methods

### 2.1. Study Population

The detailed methods have been previously published [14,15]. This study adopted a multistage, stratified, random cluster sampling scheme. From January 2012 to August 2013, a representative sample of individuals who were at 35 years of age or older was selected in order to examine the prevalence, incidence, and natural history of cardiovascular risk factors in Liaoning Province. In the first stage, three counties (Dawa, Zhangwu, and Liaoyang County) were selected randomly to represent the eastern, southern, and northern regions of Liaoning province. In the second stage, one town was randomly selected from each county (a total of three towns). In the third stage, 8–10 rural villages from each town were randomly selected (a total of 26 rural villages). Participants that were pregnant, had malignant tumors, malnutrition, hepatic disease, and or mental disorders were excluded from the present study. All eligible permanent residents aged ≥35 years from each village were invited to participate in the study (a total of 14,016 potential participants). Of those, 11,956 participants agreed to participate in the present study (response rate of 85.3%). This research was approved by the Ethics Committee of China Medical University (Shenyang, China, AF-SDP-07-1, 0-01). Procedures were performed in accordance with ethical standards. Written consent was obtained from each participant, after they had been informed of the objectives, benefits, medical items, and confidentiality agreement of personal information. If the participants were illiterate, we obtained written informed consents from their close relatives. We used baseline data in this report, and only participants who had complete data sets for the variables analyzed were included, yielding a final sample size of 11,039 individuals (4997 males and 6042 females).

### 2.2. Data Collection and Measurements

Via an interview with a standardized questionnaire, data was obtained on demographics, lifestyle, dietary habits, and history of disease. Using the questionnaire, the information of “any self-reported medication used in the past two weeks” was recorded including any medication used over the past two weeks by the participants. Blood pressure was measured three times at 2-min intervals after at least 5 min of rest using a standardized automatic electronic sphygmomanometer (HEM-907; Omron, Tokyo, Japan) according to American Heart Association protocol, which had been validated according to the British Hypertension Society protocol. The participants were advised to avoid caffeinated beverages and exercise for at least 30 min before the measurement. During the measurement, the participants were seated with their arm supported at the level of the heart. The mean of three blood pressure (BP) measurements was calculated and used in all analyses. Blood samples were obtained from all of the subjects in the morning after at least 12 h of fasting. Serum uric acid, as well as other routine blood biochemical indexes, were analyzed using an auto-analyzer (Olympus AU 640; Olympus Corp., Kobe, Japan). Details of data collection and measurements methods of this study were described elsewhere [16,17].

### 2.3. Alcohol Consumption Assessment

Alcohol consumption was assessed during the interview using the questionnaire. Participants were asked to provide information regarding whether they regularly consumed alcohol, their average alcohol consumption per day, and the number of days per month that they consumed alcohol. The ethanol weight content differed among beverages: 5% for beer, 12.5% for red wine, and 45% for hard liquor. One drink was defined as an average of 15 g of ethanol [18]. We used cut-off values based on the definition of daily alcohol consumption from the National Institute on Alcohol Abuse and Alcoholism to classify the participants’ level of consumption: non-drinkers (abstainers, no alcohol consumption history), moderate drinkers (up to 1 drink/day for women and up to 2 drinks/day for men), and heavy drinkers (>1 drink/day for women and >2 drinks/day for men) [19].

### 2.4. Definitions

Hyperuricemia was defined as serum uric acid ≥416 μmol/L in men and ≥357 μmol/L in women according to guidelines [20]. According to the Seventh Report of the Joint National Committee, hypertension is defined as a systolic BP (SBP) above 140 mm Hg and/or a diastolic BP (DBP) above 90 mm Hg and/or use of antihypertensive medications [21]. According to the World Health Organization (WHO) criteria, obese was defined as body mass index (BMI) values ≥30 kg/m^2^ [12]. Dyslipidemia was determined by the National Cholesterol Education Program, Third Adult Treatment Panel criteria [22]. Fasting plasma glucose (FPG) above 7 mmol/L (126 mg/dL) and/or receiving treatment for diabetes was diagnosed as diabetes according to the WHO criteria [23]. Using the Chronic Kidney Disease Epidemiology Collaboration equation, the glomerular filtration rate (GFR) was estimated [24]. Decreased eGFR was defined as an estimated GFR (eGFR) <60 mL/min/1.73 m^2^ [25].

### 2.5. Statistical Analysis

As described in detail previously [16,17], descriptive statistics were calculated for all the variables, including continuous variables (expressed as the mean values and standard deviations) and categorical variables (expressed as numbers and proportions). Differences among categories were evaluated using a *t*-test, an ANOVA, non-parametric tests or a χ^2^ test, as appropriate. Comparisons between groups were performed using the Scheffe method. Multivariate logistic regression analyses were used to identify independent associations between weight, metabolic status, and prolonged QTc in different models by calculating the odds ratios (ORs) and corresponding 95% confidence intervals (CIs). All of the statistical analyses were performed using SPSS version 20.0 software, and *p* values less than 0.05 were considered to be statistically significant.

## 3. Result

### 3.1. Baseline Characteristics of the Study Population by Gender

The baseline characteristics of the included participants are shown in Table 1. In total, 11,039 participants aged 35 years or older were included (4997 men and 6042 women). Male participants had a higher rate of current drinking than females (44.9% vs. 2.90%, *p* < 0.001). The percentage of non-, moderate, and heavy drinkers in males was 50.5%, 15.0%, and 34.5%, respectively; the percentage of non-, moderate, and heavy drinkers in females was 96.8%, 1.2%, and 2.0%, respectively. The mean level of uric acid was 333.3 ± 83.5 mmol/L in males and 255.8 ± 67.7 mmol/L in females. The total mean alcohol consumption per day was also lower in females than in males (1.0 ± 8.3 g/d vs. 32.6 ± 49.6 g/d, *p* < 0.001). Alcohol consumption from beer was 3.9 ± 12.8 g/d for males and 0.1 ± 2.0 g/d for females. Alcohol consumption from liquor was 28.6 ± 46.9 g/d for males and 0.9 ± 7.7 g/d for females. Alcohol consumption from wine was 0.0 ± 1.8 g/d for males and 0.0 ± 0.0 g/d for females.

### 3.2. Levels of Alcohol Consumption and Serum Uric Acid

**Figure 1:**
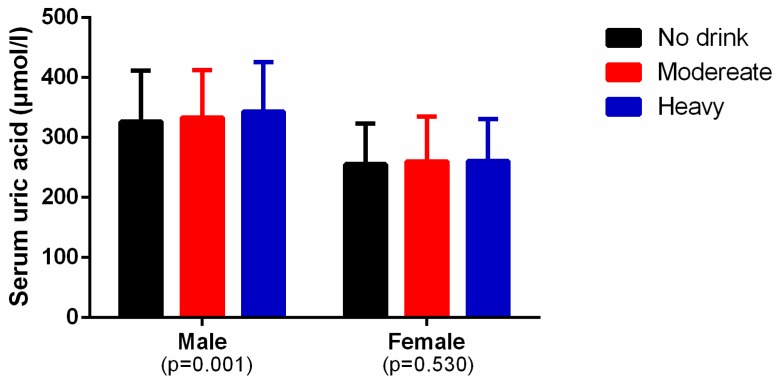
The mean level of serum uric acid in different alcohol consumption groups by gender.

**Figure 2:**
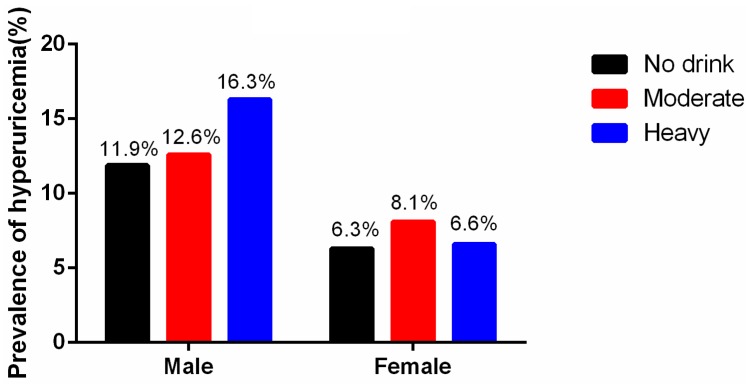
Prevalence of hyperuricemia in different alcohol consumption levels by gender.

**Figure 3:**
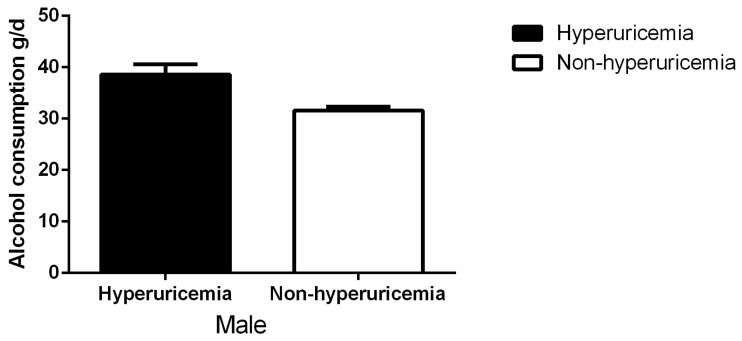
Alcohol consumption by hyperuricemia or not in males.

**Figure 4:**
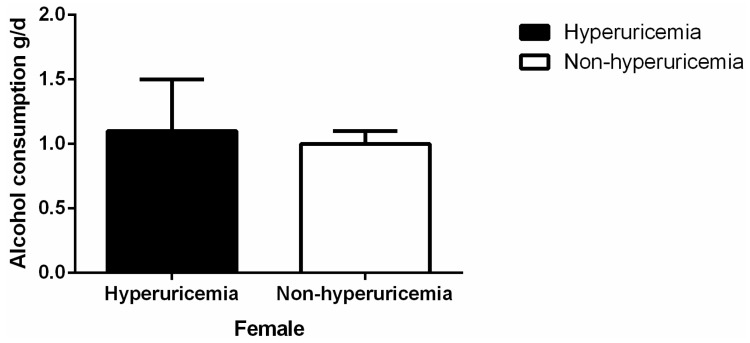
Alcohol consumption by hyperuricemia or not in females.

As shown in Figure 1, among the males, the mean level of serum uric acid was higher in heavy and moderate drinkers than in the non-drinkers (heavy vs. non-drinker: 343.0 ± 83.2 vs. 326.6 ± 84.7 mmol/L, *p* < 0.001; moderate vs. non-drinker: 333.4 ± 78.8 vs. 326.6 ± 84.7, *p* = 0.027). Among the females, there was no difference among the three groups. Figure 2 shows the prevalence of hyperuricemia in the different male alcohol consumption groups was 11.9% in non-drinkers, 12.6% in moderate drinkers, and 16.3% in heavy drinkers (*p* < 0.001). In females, the rates were 6.3% in non-drinkers, 8.1% in moderate drinkers, and 6.6% for heavy drinkers (*p* = 0.818). Alcohol consumption was much higher in males with hyperuricemia than in those without hyperuricemia (38.6 ± 2.0 g/d vs. 31.6 ± 0.75 g/d, *p* = 0.001) (Figure 3). Alcohol consumption was slightly higher in females with hyperuricemia than in those without hyperuricemia; however, the difference was not significant (1.1 ± 0.4 g/d vs. 1.0 ± 0.1 g/d, *p* = 0.530) (Figure 4).

### 3.3. Association Between Alcohol Consumption and Hyperuricemia by Gender

**Figure 5:**
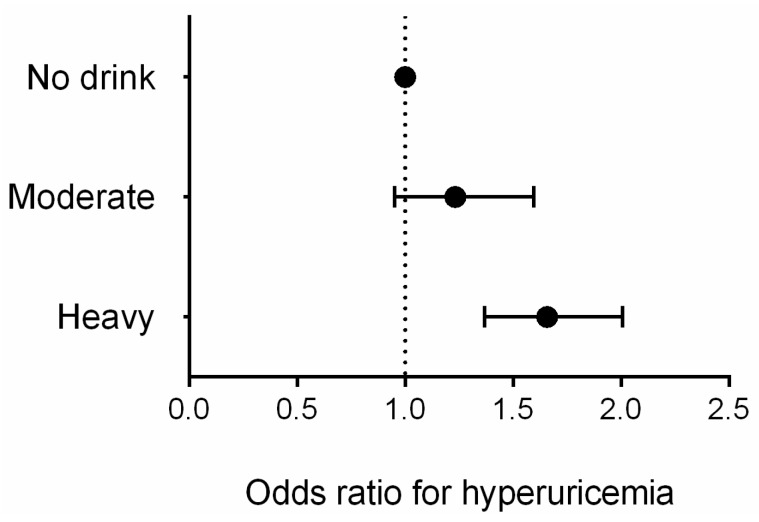
Multivariate logistic regression analyses of the risk of hyperuricemia corrected according to the different levels of alcohol consumption in males.

**Figure 6:**
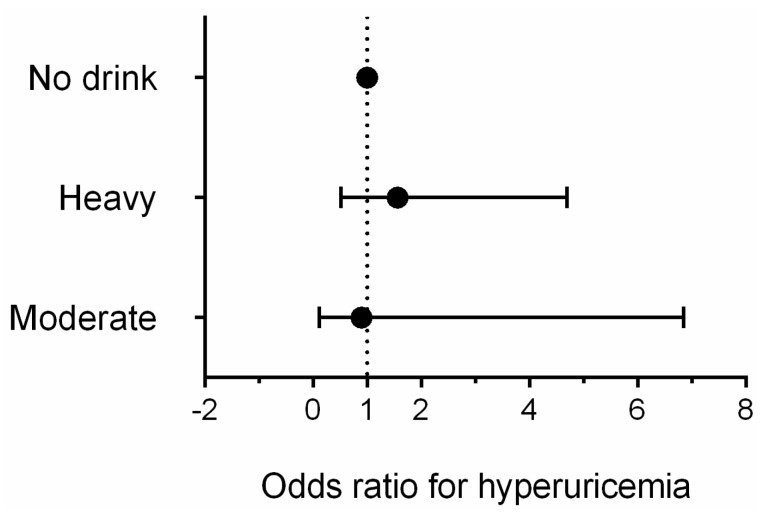
Multivariate logistic regression analyses of the risk of hyperuricemia corrected according to the different levels of alcohol consumption in females.

Table 2 presents characteristics of study population according to homocysteine or not by gender. Similar to the previous reports [26], the difference of age, life styles, BMI, blood pressure, eGFR, lipid level, prevalence of disease, and medication used was significant between hyperuricemia and not hyperuricemia. Additionally, post-menopausal females had higher prevalence of homocysteine than pre-menopausal females (*p* < 0.001). So we selected related variables mentioned above as the confounders in the multivariate logistic regression model. Figure 5 and Figure 6 showed multivariate logistic regression analyses of the risk of hyperuricemia corrected according to the different levels of alcohol consumption. In males, after adjusting for factors including age, race, education, income, diet scores, smoking status, activity level, obesity, hypertension, dyslipidemia, diabetes, and medication used, heavy drinkers had an approximately 1.7-fold higher risk of hyperuricemia (OR: 1.657, 95% CI: 1.368 to 2.007, *p* < 0.001) than non-drinkers; moderate drinkers did not experience a significant increase in risk (OR: 1.232, 95% CI: 0.951 to 1.596, *p* = 0.114). For females, we adjusted menopausal status plus the same factors as the males, multivariate logistic regression analyses showed that compared with non-drinkers, neither moderate nor heavy drinkers had a significantly increased risk of hyperuricemia (OR: 1.565, 95% CI: 0.521 to 4.695, *p* = 0.425 for heavy drinkers; OR: 0.897, 95% CI: 0.117 to 6.855, *p* = 0.916 for moderate drinkers).

## 4. Discussion

In the present study, we first evaluated the association between different levels of alcoholic drink consumption and hyperuricemia in a general population. We found that males who were heavy drinkers had an increased risk of hyperuricemia, but the same result was not found in females who were heavy drinkers. In both males and females, moderate alcohol consumption, compared with the absence of alcohol consumption, did not increase the risk of hyperuricemia.

Hyperuricemia is considered the precursor to gout, which can cause multiple organ damage [27,28,29,30,31]. Although the relationship between uric acid and drinking has been well investigated, there is a lack of research specifically focusing on different levels of alcohol consumption and hyperuricemia. A study including 2062 apparently healthy volunteers from Finland reported that serum uric acid concentrations in male moderate drinkers were significantly higher than those in the corresponding groups of alcohol abstainers, and in females, these concentrations were lower [12]. A study consisting of Japanese men who worked for a metal products factory in Toyama prefecture, between the ages of 20–54 years, reported that both moderate and heavy alcohol intake increased the risk of incident hyperuricemia [32]. Our previous studies also focused on drinking and hyperuricemia; however, the effect of different levels of alcohol consumption has not been studied [33]. To the best of our knowledge, the present study is the first to focus on the relationship between different levels of alcohol consumption and hyperuricemia in a general population in rural China. The results show that heavy but not moderate drinkers had an increased risk of hyperuricemia in males. Compared with the absence of alcohol consumption, moderate alcohol consumption did not increase the risk of hyperuricemia in both males and females. It is possible that the results are affected in part by the menopausal status the female cohort since estrogen is known to increase renal uric acid clearance [34]. Additionally, in this study, alcohol consumption from beer in males was significantly higher than in woman population. Since beer contains large amounts of purine, it is possible that attenuated rise in serum uric acid levels in the male cohort was due to the differences in beer consumption. So we adjusted bottles of beer consumption per week in the multivariate analyses for further analyses and got the same conclusions which data was shown in Table 3.

However, the mechanism of the different effects due to different levels of alcohol consumption is unclear. We speculate that moderate alcohol intake can increase the glomerular filtration rate, which may increase uric acid excretion. However, this requires further study. In addition, we found that males who were heavy drinkers had an increased risk of hyperuricemia, while the same result was not observed in females. There are several reasons for these disparate results. First, in this general population, there were significantly fewer female drinkers than male drinkers; therefore, we speculate that the smaller sample size may have driven the non-significant results. Second, women absorb and metabolize alcohol differently than men [35,36]. However, further research should be conducted to explore the mechanism underlying our findings.

This estimate has an important implication for public health policy. Our present results corroborate previous findings that moderate alcohol consumption was reasonable for men and women. For an individual person, hyperuricemia could exist in combination with heart disease and other diseases, as shown in our basic characteristics. However, other studies have reported that moderate consumption of alcohol is harmless for some diseases such as coronary heart disease and ischemic stroke [37,38]. As such, according to the present results, a reduction in alcohol intake in heavy drinkers among the male population could be recommended to lower the risk of hyperuricemia.

Our study had some limitations. First, the data are not representative of the adult population throughout China. Second, since some of the study population had several risk factors including hypertension, DM, and dyslipidemia, we could not eliminate the possible effects of underlying diseases and medications used for these diseases on the present findings. Additionally, we did not analyze every type of medication used by the participants, including uric acid lowering medication which may affect the results, but we adjusted the “any self-reported medication used in the past two weeks” in the multivariate analyses which may reduce the influence of drug confounders. Finally, our study was descriptive and the results were obtained using a cross-sectional design, thus, the conclusions are based only on correlations and no cause-and-effect relationships could be established. So, further prospective population-based studies are needed to confirm our result and investigate the related mechanism.

## 5. Conclusions

In conclusion, based on this rural general population, we found that male heavy drinkers had increased odds of hyperuricemia, but the same result was not found in females. Among both males and females, moderate alcohol consumption did not increase the odds of hyperuricemia. This will have practical implications for prevention and management of hyperuricemia in rural areas.

## Figures and Tables

**Table 1:** Baseline characteristics of study population by gender (*N* = 11039).

Variables	Male (4997)	Female (6042)	*p*-value
Age (year)	54.4 ± 10.8	53.4 ± 10.3	<0.001
Current drinking	2244 (44.9%)	173 (2.9%)	<0.001
Total alcohol consumption (g/d)	32.6 ± 49.6	1.0 ± 8.3	<0.001
Alcohol consumption from beer (g/d)	3.9 ± 12.8	0.1 ± 2.0	<0.001
Alcohol consumption from liquor (g/d)	28.6 ± 46.9	0.9 ± 7.7	<0.001
Alcohol consumption from wine (g/d)	0.0 ± 1.8	0.0 ± 0.0	<0.001
Alcohol consumption			<0.001
No drink	2524 (50.5%)	584 7 (96.8%)	
Moderate	751 (15.0%)	74 (1.2%)	
Heavy	1722 (34.5%)	121 (2.0%)	

Data are expressed as the mean ± SD or as n (%).

**Table 2:** Baseline characteristics of study population according to hyperuricemia or not by gender.

Variables	Female			Male		
	Non-Hyperuricemia	Hyperuricemia	*p*-value	Non-Hyperuricemia	Hyperuricemia	*p*-value
Age (year)	53.1 ± 10.2	57.8 ± 10.6	<0.001	54.6 ± 10.8	53.2 ± 11.1	0.002
SBP (mmHg)	139.7 ± 2.8	146.4 ± 25.0	<0.001	143.2 ± 22.6	145.6 ± 22.9	0.010
DBP(mmHg)	80.3 ± 11.4	83.9 ± 12.7	<0.001	83.1 ± 11.6	87.2 ± 12.7	<0.001
Serum creatinine (mmol/L)	64.3 ± 11.8	82.7 ± 63.1	<0.001	78.1 ± 16.2	88.7 ± 30.3	<0.001
eGFR(mL/min)	95.2 ± 14.3	88.0 ± 19.4	<0.001	92.9 ± 15.4	75.6 ± 19.1	<0.001
Serum uric acid (μmol/L)	245.1 ± 53.7	413.8 ± 54.7	<0.001	309.9 ± 58.3	482.9 ± 62.6	<0.001
FPG (mmol/L)	5.8 ± 1.6	6.2 ± 1.6	<0.001	5.9 ± 1.7	6.0 ± 1.5	0.705
TC (mmol/L)	5.3 ± 1.1	5.8 ± 1.3	<0.001	5.1 ± 1.0	5.5 ± 1.2	<0.001
TG (mmol/L)	1.6 ± 1.2	2.5 ± 2.2	<0.001	1.5 ± 1.4	2.5 ± 2.7	<0.001
HDL-C (mmol/L)	1.4 ± 0.3	1.3 ± 0.3	<0.001	1.4 ± 0.4	1.3 ± 0.4	<0.001
LDL-C (mmol/L)	2.9 ± 0.8	3.3 ± 0.9	<0.001	2.8 ± 0.8	3.0 ± 0.8	<0.001
Dietscore	2.1 ± 1.1	2.0 ± 1.1	0.009	2.5 ± 1.1	2.6 ± 1.1	0.101
BMI(kg/m^2^)	24.7 ± 3.7	26.7 ± 3.8	<0.001	24.5 ± 3.4	26.4 ± 3.9	<0.001
Race group (han)	5361 (94.8%)	374 (97.4%)	0.010	4092 (94.7%)	646 (95.6%)	0.200
Education			<0.001			0.535
Primary school or below	3173 (56.1%)	258 (67.2%)		1811 (41.9%)	278 (41.1%)	
Middle school	2047 (36.2%)	97 (25.3%)		2036 (47.1%)	314 (46.4%)	
High school or above	438 (7.7%)	29 (7.6%)		474 (11.0%)	84 (12.4%)	
Physical activity			0.010			0.001
Low	3173 (56.1%)	374 (97.4%)		913 (21.1%)	180 (26.6%)	
Moderate	2047 (36.2%)	10 (2.6%)		3160 (73.1%)	471 (69.7%)	
High	438 (7.7%)	384 (100.0%)		248 (5.7%)	25 (3.7%)	
Currently smoking	929 (16.4%)	66 (17.2%)	0.694	2504 (57.9%)	351 (51.9%)	0.002
Family income (CNY/year)			0.360			0.482
≤5000	646 (11.4%)	53 (13.8%)		582 (13.5%)	90 (13.3%)	
5000–20,000	3135 (55.4%)	209 (54.4%)		2349 (54.4%)	353 (52.2%)	
>20,000	1877 (33.2%)	122 (31.8%)		1390 (32.2%)	233 (34.5%)	
Medication used ^a^			0.004			<0.001
Yes	2277 (87.8%)	317 (12.2%)		2418 (96.6%)	85 (3.4%)	
No	2008 (85.0%)	355 (15.0%)		3196 (91.5%)	295 (8.5%)	
Menopause status						<0.001
Yes	–	–		3233 (91.9%)	285 (8.1%)	
No	–	–		2385 (96.2%)	94 (3.8%)	

Data are expressed as the mean ± SD or as n (%); QTc: corrected QT; CNY: China Yuan (1 CNY = 0.157 USD); BMI: body mass index; WC: waist circumference; SBP: systolic blood pressure; DBP: diastolic blood pressure; FPG: fasting plasma glucose; TC: total cholesterol; TG: triglyceride; LDL-C: low-density lipoprotein cholesterol; HDL-C: high-density lipoprotein cholesterol; ^a^ Indicating any self-reported medication used in the past two weeks; ps: *p* for category from chi-square; for continous from non-parametric test.

**Table 3:** Multivariable logistic regression analyses for hyperuricemia.

Variables	Male		Female	
	*p*-value	OR (95% CI)	*p*-value	OR (95% CI)
Race (han)	0.204	0.770 (0.515–1.152)	0.069	0.457 (0.196–1.061)
Current smoking	0.002	0.756 (0.634–0.902)	0.038	0.656 (0.440–0.977)
Medication use ^a^	0.022	1.224 (1.030–1.454)	<0.001	1.734 (1.273–2.362)
Age (years)	<0.001	0.978 (0.969–0.988)	0.308	1.009 (0.992–1.026)
Diabetes	0.058	0.760 (0.572–1.009)	0.012	1.563 (1.105–2.210)
Dyslipidemia	<0.001	2.167 (1.822–2.577)	<0.001	2.630 (1.995–3.468)
Obesity	<0.001	2.066 (1.569–2.719)	0.001	1.886 (1.303–2.730)
Hypertension	0.004	1.315 (1.094–1.581)	0.023	1.422 (1.051–1.924)
decreased eGFR	<0.001	7.400 (4.581–11.953)	<0.001	11.136 (6.873–18.042)
Annual income (CNY/year)				
≤5000	0.864	1.000 (reference)		1.000 (reference)
5000–20,000	0.589	0.929 (0.711–1.214)	0.753	1.071 (0.698–1.643)
>20,000	0.679	0.940 (0.703–1.258)	0.495	1.179 (0.735–1.890)
Dietscore	0.354	1.038 (0.959–1.123)	0.619	0.970 (0.859–1.095)
Physical activity				
Low	0.013	1.000 (reference)		1.000 (reference)
Moderate	0.047	0.811 (0.659–0.997)	0.050	1.340 (0.999–1.795)
High	0.007	0.535 (0.339–0.845)	0.991	0.996 (0.523–1.899)
Education level				
Primary school or below	0.733	1.000 (reference)	0.659	1.000 (reference)
Middle school	0.442	0.927 (0.764–1.124)	0.425	0.874 (0.628–1.217)
High school or above	0.880	0.978 (0.732–1.306)	0.813	1.068 (0.621–1.835)
Beer (bottles/weeks)	0.222	1.010 (0.994–1.027)	0.088	1.135 (0.981–1.312)
Alcohol consumption group				
No	<0.001	1.000 (reference)	0.979	1.000 (reference)
Moderate	0.168	1.201 (0.926–1.558 )	0.843	0.814 (0.106–6.238)
Heavy	<0.001	1.573 (1.285–1.926)	0.951	0.956 (0.228–4.001)
Menopause	--		0.595	1.083 (0.806–1.456)

OR: odds ratio; 95% CI: 95% confidence interval; CNY: China Yuan (1CNY = 0.157 USD); ^a^ Indicating any self-reported medication used in the past two weeks.
